# PhoP/PhoQ Two-Component System Contributes to Intestinal Inflammation Induced by *Cronobacter sakazakii* in Neonatal Mice

**DOI:** 10.3390/foods13172808

**Published:** 2024-09-04

**Authors:** Yan Ma, Yingying Zhang, Yuting Wang, Zhu Qiao, Yingying Liu, Xiaodong Xia

**Affiliations:** 1 School of Biological and Food Processing Engineering, Huanghuai University, Zhumadian 463000, China; myan9102@163.com (Y.M.); yutingwbio@163.com (Y.W.); qiaozhu2009@163.com (Z.Q.); yolanda2011yy@126.com (Y.L.); 2 The College of Life Sciences, Northwest University, Xi’an 710068, China; zhangyingyingsci@163.com; 3 State Key Laboratory of Marine Food Processing and Safety Control, National Engineering Research Center of Seafood, School of Food Science and Technology, Dalian Polytechnic University, Dalian 116034, China

**Keywords:** *Cronobacter sakazakii*, PhoP/PhoQ system, neonatal mice, intestinal inflammation

## Abstract

*Cronobacter sakazakii* (*C. sakazakii*) is a foodborne pathogen capable of causing severe infections in newborns. The PhoP/PhoQ two-component system exerts a significant influence on bacterial virulence. This study aimed to investigate the impact of the PhoP/PhoQ system on intestinal inflammation in neonatal mice induced by *C. sakazakii*. Neonatal mice were infected orally by *C. sakazakii* BAA-894 (WT), a *phoPQ*-gene-deletion strain (Δ*phoPQ*), and a complementation strain (Δ*phoPQ^C^*), and the intestinal inflammation in the mice was monitored. Deletion of the *phoPQ* gene reduced the viable count of *C. sakazakii* in the ileum and alleviated intestinal tissue damage. Moreover, caspase-3 activity in the ileum of the WT- and Δ*phoPQ^C^*-infected mice was significantly elevated compared to that of the Δ*phoPQ* and control groups. ELISA results showed elevated levels of TNF-α and IL-6 in the ileum of the mice infected with WT and Δ*phoPQ^C^*. In addition, deletion of the *phoPQ* gene in *C. sakazakii* resulted in a down-regulation of inflammatory genes (IL-1β, TNF-α, IL-6, NF-κB p65, TLR4) within the ileum and decreased inflammation by modulating the TLR4/NF-κB pathway. It is suggested that targeting the PhoP/PhoQ two-component system could be a potential strategy for mitigating *C. sakazakii*-induced neonatal infections.

## 1. Introduction

Necrotizing enterocolitis (NEC) is a prevalent and severe gastrointestinal disease observed in newborns, characterized by symptoms such as decreased appetite, weight loss, intestinal mucosal necrosis, and inflammatory infiltration [1,2]. Contributing factors to NEC encompass prematurity, formula feeding, abnormal bacterial colonization in immature intestines, and the absence of appropriate colonization by symbiotic gut microbiota [3,4]. The gut, being the largest immune organ, maintains a balance between pathogenic and symbiotic bacteria [5]. Disruptions in intestinal microbiota and infections by harmful pathogens can result in NEC [6].

*C. sakazakii*, a foodborne pathogen, can cause infections such as NEC, bacteremia, and meningitis in infants. *C. sakazakii* possesses the capability to traverse the host intestinal epithelial barrier, gain access to the bloodstream, and translocate across the blood–brain barrier, which is critical for causing severe infections [7]. Although the incidence of diseases caused by *C. sakazakii* is relatively low, the mortality rate can be as high as 40–80%, with some infants experiencing sequelae such as delayed brain development after being cured [8]. *C. sakazakii* exhibits strong resistance to various stressful environments, posing a persistent risk even after process like heat dehydration and pasteurized powdered formula production, marking infant formula a common vehicle for transmission [9]. 

The two-component system (TCS) represents a fundamental mechanism of bacterial signal transduction, enabling bacteria to respond to environments [10]. A typical TCS comprises a histidine kinase sensor (HK) and a response regulator (RR) [11]. Several TCSs exert pivotal regulatory functions in bacterial virulence [12]. The QseB/QseC TCS is crucial for bacterial virulence, drug resistance, and environmental tolerance [13]. The PhoP-PhoR TCS is essential for *Mycobacterium tuberculosis* virulence [14]. OmpR was critical for the virulence and infection of *Klebsiella pneumoniae* in a murine model of pulmonary infection [15]. The QseBC TCS has been implicated in the virulence, motility, and metabolic regulation of various Gram-negative pathogens [16].

The PhoP/PhoQ TCS modulates Mg^2+^ homeostasis, environmental tolerance, antimicrobial resistance, and toxicity in various bacteria [17]. It has been reported that the PhoP/PhoQ TCS enhances the tolerance of *E. coli* O157:H7 to acidity and heat under weakly acidic conditions [18]. Changes in the PmrA/PmrB or PhoP/PhoQ systems have been recognized as a key driver contributing to elevated levels of resistance against colistin and polymyxin in cystic fibrosis patients [19]. In *Salmonella*, the PhoP/PhoQ TCS plays a crucial role in the regulation of virulence [20]. Despite extensive studies on the various regulatory functions of the PhoP/PhoQ TCS, the mechanism in the pathogenesis of *C. sakazakii* remains unclear.

*C. sakazakii* infection models in neonatal mice are frequently utilized to investigate the inflammatory mechanisms of neonatal infections [21]. In this study, the *C. sakazakii* BAA-894 strain, along with *phoPQ*-gene-deletion and complemented strains, were used to infect newborn mice to construct a model of *C. sakazakii*-induced intestinal inflammation. This study investigated the effect of *phoPQ* gene deletion on intestinal damage caused by *C. sakazakii* in neonatal mice. Immunohistochemical staining, caspase-3 activity detection, ELISA, qRT-PCR, and Western blot analyses were used to study the impact of *phoPQ* gene deletion on the expression of inflammatory factors in neonatal mouse ileum tissue following infection.

## 2. Materials and Methods

### 2.1. Strains

The WT strain was *C. sakazakii* BAA-894/NA (nalidixic acid (NA) resistance was induced for gene knockout). The Δ*phoPQ* strain (*phoPQ* gene knockout of BAA-894) and Δ*phoPQ^C^* (complementation strain) were constructed as previously described [22]. The WT and Δ*phoPQ* strains were grown in Luria–Bertani (LB) medium containing 32 μg/mL NA 37 °C, and the Δ*phoPQ* strain was cultured in LB medium containing 32 μg/mL NA and 20 μg/mL chloramphenicol at 37 °C. 

### 2.2. Animal Experiment

Twenty male and female CD-1 mice (specific-pathogen-free) were purchased from Chengdu Dossy (Chengdu, China) (SCXK 2020-0030). All animal experiments were conducted in accordance with the Guide for the Care and Use of Laboratory Animals: Eighth Edition (ISBN-10:0-309-15396-4) and approved by the Animal Ethics Committee of Dalian Polytechnic University (DLPU2022082). Each cage was randomly divided into one male and one female mouse. Feeding conditions were as follows: temperature 23 ± 3 °C, humidity 60–70%, light and dark alternating for 12 h, and free water feeding. Female mice were fed separately after pregnancy and then randomly grouped according to cage after natural delivery. Neonatal mice were raised with their mothers after birth and randomly allocated into 4 groups (*n* = 30): (1) PBS (control); (2) WT; (3) Δ*phoPQ*; and (4) Δ*phoPQ^C^*. Neonatal mice were subjected to induction of intestinal inflammation according to a previous method with slight modifications [23]. In brief, 3-day-old pups were treated by oral gavage, in which each control pup was given sterile PBS, and the experimental mice were given PBS containing 10^7^ CFU WT, Δ*phoPQ,* and Δ*phoPQ^C^* strains, respectively. After treatment, the mice were weighed daily, and morbidity was observed. At postnatal day 10, all mice were euthanized, and intestinal tissues were subsequently collected.

### 2.3. Number of C. sakazakii

Fresh ileum tissues were prepared into 10% tissue homogenate using pre-cooled sterile PBS solution (1:10, *w*/*v*). After serial dilution, 100 μL of each tissue solution was inoculated onto a chromogenic medium of *C. sakazaki*, incubated at 37 °C for 24 h. The results are represented by “CFU/mg ileum”.

### 2.4. Histopathology

The intestinal tissues were fixed overnight with 4% paraformaldehyde and paraffin-embedded. The sections were stained by H&E and analyzed by a light microscope (Leica, Wetzlar, Germany). The intestinal histopathology was scored as follows: histopathological scores were performed according to the degree of epithelial shedding, inflammatory cell infiltration, and tissue structure destruction as follows: normal (0); slight (1); moderate (2); severe (3); necrosis (4) [24].

### 2.5. Caspase-3 Activity

The ileum tissue of 5–6 newborn mice in each group was mixed, and caspase-3 activity was detected by a kit (Beyotime, Shanghai, China). The ileum tissues and the lysate were ground on ice and then centrifuged (12,000× *g*, 15 min, 4 °C). The protein concentration in the supernatant was measured with a Bradford Protein detection Kit (Beyotime). After mixing the sample with the buffer solution, the caspase-3 colorimetric substrate Ac-DEVD-pNA was added and incubated at 37 °C for 2 h, and then the OD_405_ was measured.

### 2.6. ELISA

The ileum tissues of mice in each group were homogenized with the pre-cooled PBS solution at a ratio of 1:9 (weight: volume) and then centrifuged (5000× *g*, 10 min, 4 °C), and the supernatant was retained. Levels of TNF-α and IL-6 in the ileum were measured by an ELISA kit (Ameko, Shanghai, China).

### 2.7. qRT-PCR

Total RNA was extracted by a Steady Pure RNA extraction kit (AG, Changsha, China), followed by reverse transcription into cDNA by an Evo M-MLV reverse transcription kit. The qRT-PCR analysis was conducted using an IQ5 system (Bio-Rad). The primer sequence, along with GAPDH as the reference gene, is presented in Table 1.

### 2.8. Immunohistochemical Staining

The level of NF-κB p65 in the ileum was assessed using immunohistochemical staining [29]. Briefly, paraffin sections were treated with dewaxing, antigen retrieval, and blocking endogenous peroxidase, followed by a 30 min incubation with 10% rabbit serum for blocking. The sections were incubated with the primary antibody at 4 °C overnight followed by a 50 min incubation with the secondary antibody, and they were finally stained with DAB and counterstained with hematoxylin. The results were observed by fluorescence microscopy (Leica, Wetzlar, Germany), and the mean optical density was evaluated by Image J (Version 1.8.0.112).

### 2.9. Western Blot

The ileal tissues were homogenized in lysate buffer containing a mixture of phenylmethylsulfonyl fluoride, protease, and phosphatase inhibitors (Beyotime) and then centrifuged (4 °C, 12,000× *g*, 10 min). The protein of the supernatant was detected with a BCA protein assay kit (Beyotime). The protein samples were separated by 10% SDS polyacrylamide gel electrophoresis followed by transfer onto a nitrocellulose membrane. The membranes were blocked with 5% skim milk for 2 h and then incubated with the primary antibodies at 4 °C overnight and with the secondary antibodies for 2 h. The ECL reagent was employed for protein band visualization, and the results were quantified using Image J with beta-actin as an internal reference.

### 2.10. Statistical Analysis

The data are presented as means ± SD. SPSS 20.0 was used for one-way analysis of variance, and *p* < 0.05 was considered statistically significant.

## 3. Results

### 3.1. Deletion of phoPQ Gene Reduced the Count of C. sakazaki in Ileum

**Figure 1:**
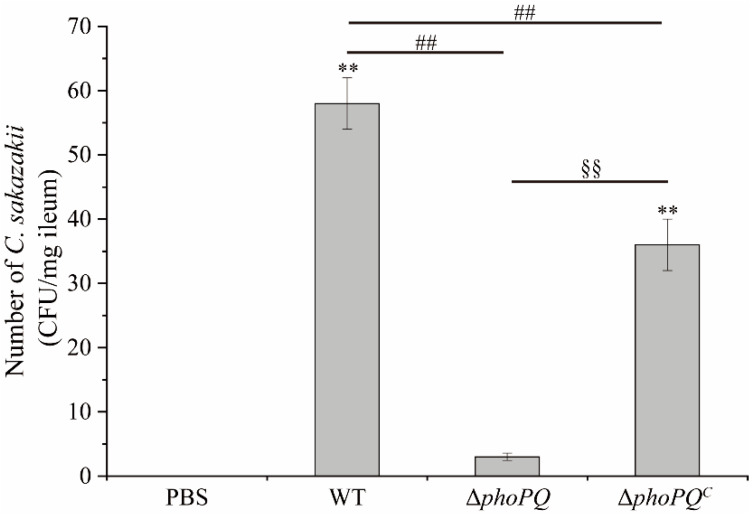
The count of *C. sakazakii* in ileum. *, #, and § represent comparisons with PBS, WT, and Δ*phoPQ* groups, respectively. ** *p*, ^##^ *p*, and ^§§^ *p* mean *p* < 0.01 (*n* = 5/group).

No *C. sakazakii* was detected in the control group (Figure 1). The counts of *C. sakazakii* colonies in the ileum of the mice in the WT group and Δ*phoPQ^C^* group were 58 ± 4 CFU/mg and 36 ± 4 CFU/mg, respectively, whereas the count significantly decreased to only 3 ± 0.57 CFU/mg in the Δ*phoPQ* group. This indicates that the *phoPQ* deletion reduced the colonization ability of *C. sakazakii* in the mice.

### 3.2. PhoP/PhoQ System Affected the Histopathological Damage

**Figure 2:**
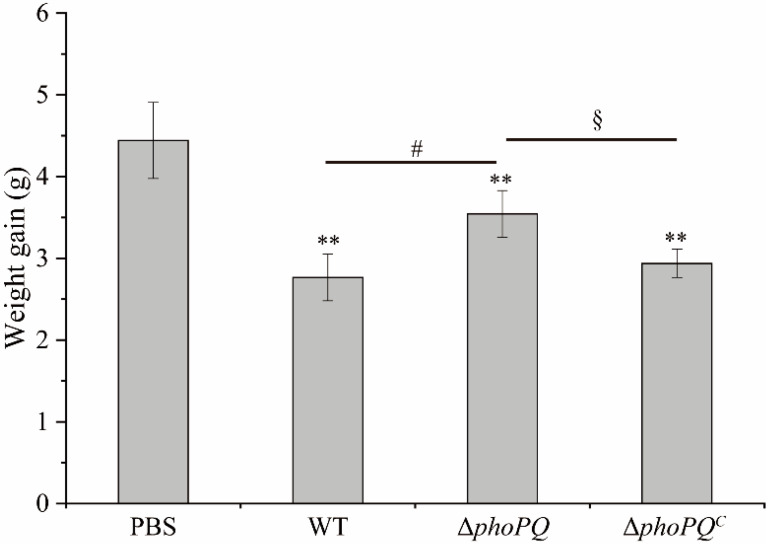
Effect of *phoPQ* gene deletion on weight gain. *, #, and § represent comparisons with PBS, WT, and Δ*phoPQ* groups, respectively. ^#^ *p*, and ^§^ *p* mean *p* < 0.05, and ** *p* mean *p* < 0.01 (*n* = 30/group).

Compared with the PBS group, the mice infected with *C. sakazakii* had slower growth from day 3 to day 10 after birth, with no mortality observed. However, the weight gain of the mice infected with the WT and Δ*phoPQ^C^* strains was significantly lower (*p* < 0.05) compared to that of the Δ*phoPQ* group (Figure 2).

**Figure 3:**
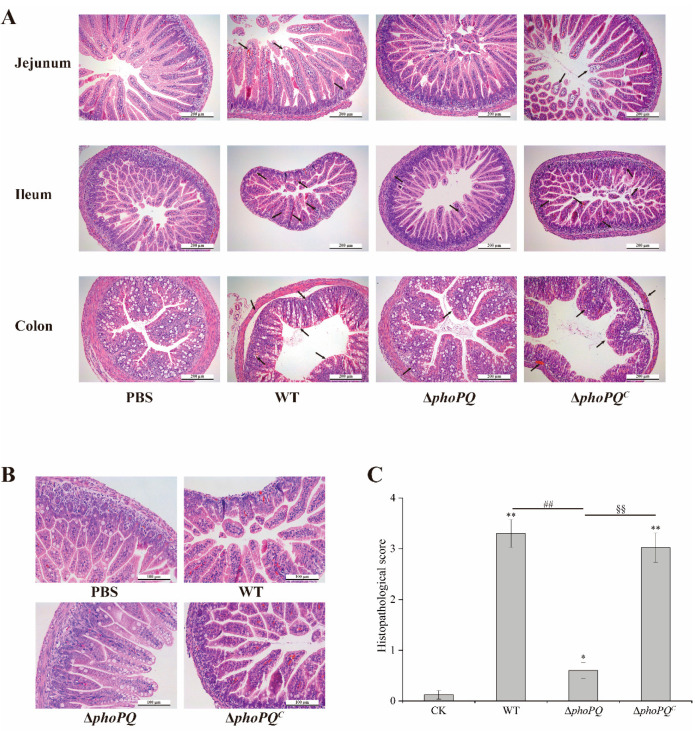
H&E staining and pathology score of neonatal mice intestinal tissues. Arrows indicate epithelial shedding, villi destruction, and goblet cell reduction. (**A**): H&E staining of jejunum, ileum, and colon tissue of neonatal mice (100×); (**B**): H&E staining of ileum tissues (200×); (**C**): histopathological score of ileum tissues. *, #, and § represent comparisons with PBS, WT, and Δ*phoPQ* groups, respectively. * *p* mean *p* < 0.05, and ** *p*, ^##^ *p*, and ^§§^ *p* mean *p* < 0.01.

The H&E staining results of the jejunum, ileum, and colon of the newborn mice in the different groups are shown in Figure 3A. The intestinal morphology of the PBS group appeared normal, with no evident inflammation and damage. The jejunal injury in the mice infected with *C. sakazakii* was mild, while ileal and colonic lesions were severe. Due to the smaller size of the intestines in neonatal mice and the intestinal contents in the colon being relatively large, which may have interfered with the subsequent experiments, the ileum tissue was selected for the follow-up studies. The ileum tissues were observed under a magnification of 200× (Figure 3B). The ileum morphology of the mice infected with the WT strain showed severe damage, including epithelial shedding, villi destruction, and goblet cell reduction. In contrast, the ileal structure in the Δ*phoPQ* group remained relatively intact with reduced lesions. The ileal lesions in the Δ*phoPQ^C^* group were more serious than those in the Δ*phoPQ* group. The histopathological scores of the three groups infected with *C. sakazakii* exhibited significantly higher values compared to those in the PBS group. Although no significant difference was observed between the WT group and Δ*phoPQ^C^* group, both groups exhibited significantly higher scores compared to the Δ*phoPQ* group (*p* < 0.05) (Figure 3C).

### 3.3. Deletion of phoPQ Gene in C. sakazakii Affected Caspase-3 Activity

**Figure 4:**
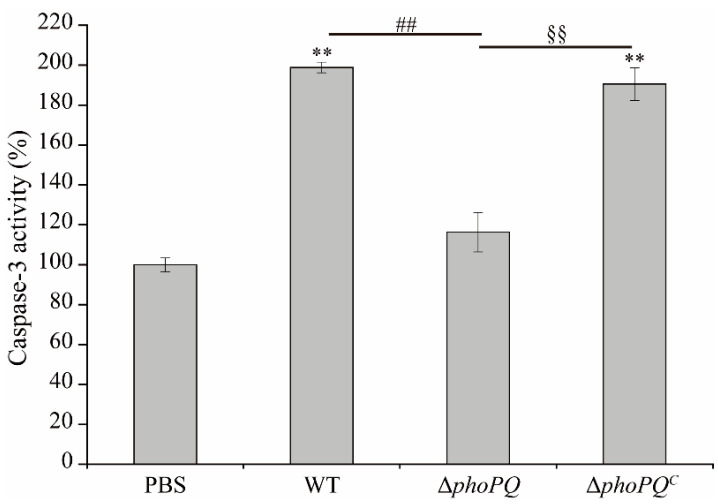
Caspase-3 activity in ileum of newborn mice in PBS, WT, Δ*phoPQ*, and Δ*phoPQ^C^* groups. *, #, and § represent comparisons with PBS, WT, and Δ*phoPQ* groups, respectively. ** *p*, ^##^ *p*, and ^§§^ *p* mean *p* < 0.01 (*n* = 5–6/group).

The caspase-3 activity in the ileum of both the WT and Δ*phoPQ^C^* groups exhibited a significant increase compared to that in the PBS group, with respective fold changes of 1.99 and 1.90. The Δ*phoPQ* and PBS groups exhibited comparable levels of capase-3 activity, indicating no significant difference between them (Figure 4).

### 3.4. Deletion of phoPQ Gene in C. sakazakii Affected TNF-α and IL-6 Levels in Ileum

**Figure 5:**
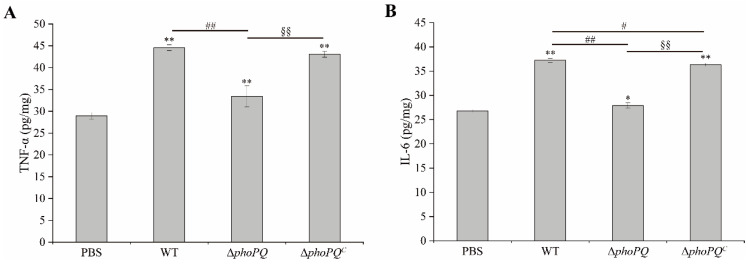
The levels of TNF-α (**A**) and IL-6 (**B**) in ileum of newborn mice in PBS, WT, Δ*phoPQ*, and Δ*phoPQ^C^* groups. *, #, and § represent comparisons with PBS, WT, and Δ*phoPQ* groups, respectively. * *p* and ^#^ *p* mean *p* < 0.05, and ** *p*, ^##^ *p*, and ^§§^ *p* mean *p* < 0.01 (*n* = 5/group).

The levels of TNF-α and IL-6 in the ileum of the mice infected with the WT strain and Δ*phoPQ^C^* strain were significantly elevated compared to those in the PBS group and Δ*phoPQ* group (*p* < 0.05). The TNF-α levels of the WT, Δ*phoPQ*, and Δ*phoPQ^C^* groups increased by 53.97%, 15.38%, and 48.72%, respectively, compared with the PBS group (Figure 5A). Similarly, the IL-6 levels in the WT, Δ*phoPQ*, and Δ*phoPQ^C^* groups increased by 38.93%, 4.14%, and 35.54%, respectively (Figure 5B). The results showed that *phoPQ* gene deletion significantly reduced the TNF-α and IL-6 levels within the ileum of the *C. sakazakii*-infected neonatal mice.

### 3.5. Deletion of phoPQ Gene Affected the Expression of Inflammation-Related Genes

**Figure 6:**
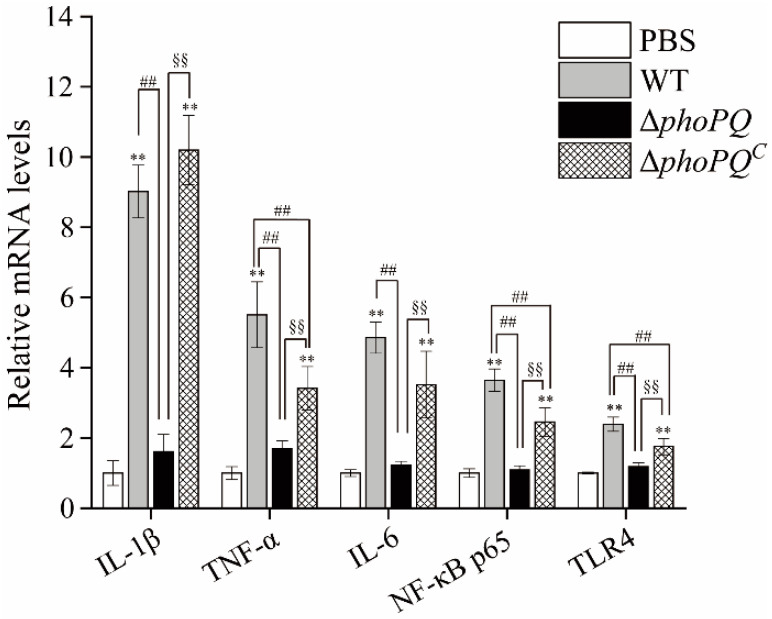
Relative mRNA levels of target genes in the ileum tissue of newborn mice by qRT-PCR. *, #, and § represent comparisons with PBS, WT, and Δ*phoPQ* groups, respectively. ** *p*, ^##^ *p*, and ^§§^ *p* mean *p* < 0.01 (*n* = 5/group).

The levels of target genes in the ileum of the neonatal mice in the four groups was detected by qRT-PCR (Figure 6). Compared to the normal mice, the mRNA transcription levels of IL-1β, TNF-α, IL-6, NF-κB p65, and TLR4 were significantly up-regulated in the ileum of the WT- and Δ*phoPQ^C^*-infected mice (*p* < 0.05). However, the expression levels of the target genes in the ileum of the Δ*phoPQ*-strain-infected mice did not exhibit significant differences compared to those in the PBS group.

### 3.6. Deletion of phoPQ Gene Affected the Expression of NF-κB p65 

**Figure 7:**
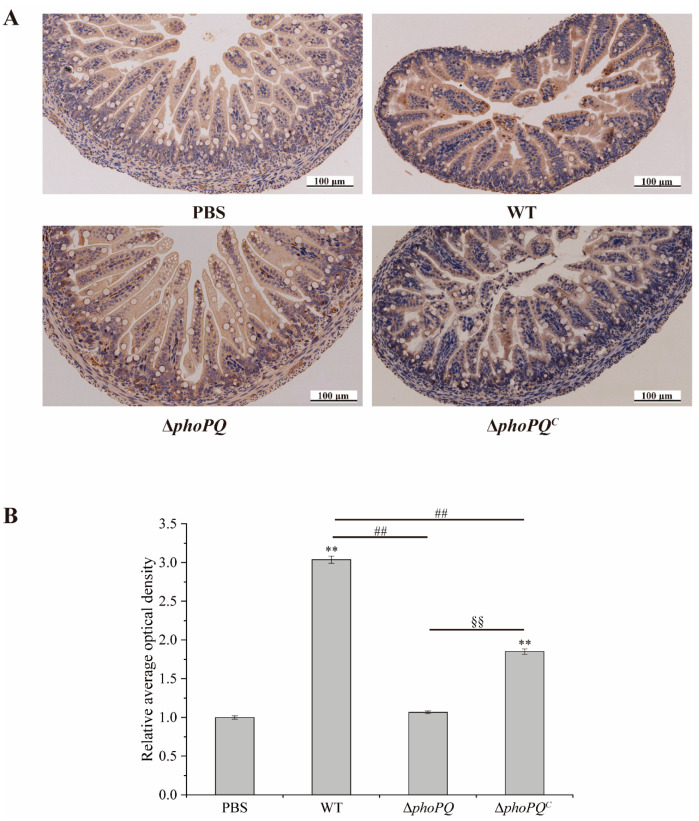
Immunohistochemical staining of NF-κB p65 protein in the ileum of newborn mice. (**A**): Representative images of immunohistochemistry; (**B**): the level of NF-κB p65 protein analyzed by relative average optical density. *, #, and § represent comparisons with PBS, WT, and Δ*phoPQ* groups, respectively. ** *p*, ^##^ *p*, and ^§§^ *p* mean *p* < 0.01.

The expression of NF-κB p65 in the ileum was measured by immunohistochemical staining (Figure 7A,B). The level of NF-κB p65 protein in the mice infected with the WT and Δ*phoPQ^C^* strains exhibited a significantly higher level compared to that of the PBS group (*p* < 0.05). However, no significant difference was observed between the mice infected with the Δ*phoPQ* strains and the PBS group.

### 3.7. Deletion of phoPQ Gene in C. sakazakii Affected the Protein Expression of IκBα and TLR4 in the Ileum

**Figure 8:**
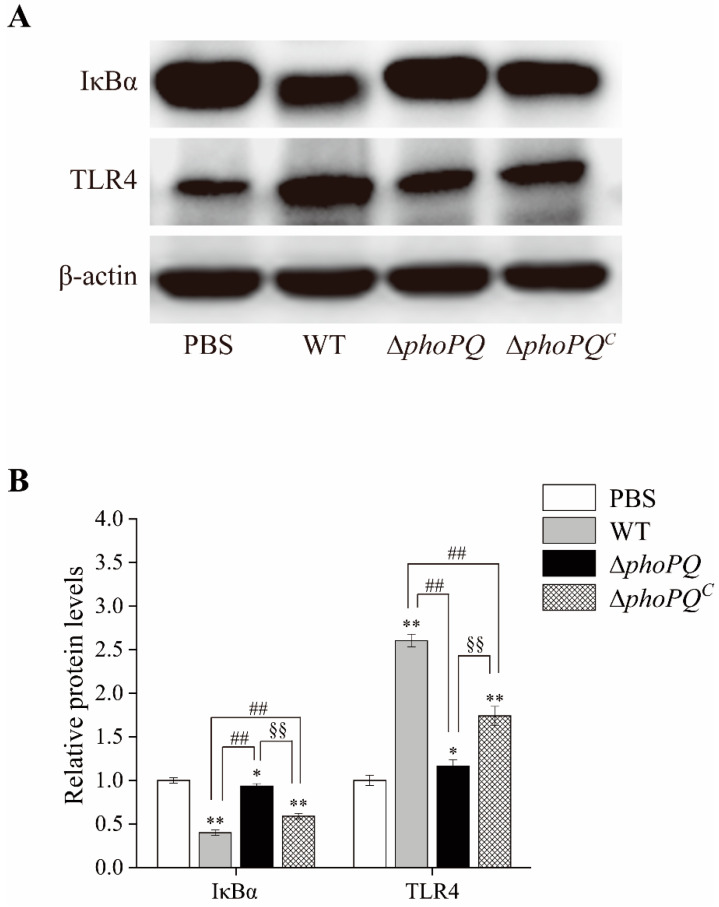
The protein levels of IκBα and TLR4 in the ileum. (**A**): Representative images; (**B**): relative protein levels of IκBα and TLR4. *, #, and § represent comparisons with PBS, WT, and Δ*phoPQ* groups, respectively. * *p* mean *p* < 0.05, and ** *p*, ^##^ *p*, and ^§§^ *p* mean *p* < 0.01 (*n* = 5/group).

Western blot analysis showed that TLR4 (toll-like receptor 4) protein expression was significantly up-regulated in the ileum of the *C. sakazakii*-infected neonatal mice compared to the control group. Moreover, the expression levels in the mice infected with the WT and Δ*phoPQ^C^* strains were significantly elevated compared to those in the Δ*phoPQ*-infected mice. In contrast, IκBα (NF-κB inhibitor protein) protein expression was down-regulated in the *C. sakazakii*-infected mice, with significant decreases observed in the WT and Δ*phoPQ^C^* groups compared to the Δ*phoPQ* group (Figure 8A,B).

## 4. Discussion

*C. sakazakii* can cause NEC in infants, with a fatality rate reaching up to 80% [30]. NEC is a high-disability, high-mortality, and devastating disease that poses a serious threat to the health of newborns [1]. Compared with BALB/c and C57BL/6 mice, CD-1 mice exhibited the highest susceptibility to *C. sakazakii*, having the lowest infecting and lethal doses. This suggests that CD-1 mice are suitable models for studying *C. sakazakii* infection in neonates [21]. Shi et al. [31] established a neonatal mouse NEC model by gavaging 3-day-old mice with 1 × 10^7^ CFU *C. sakazakii* ATCC29544, demonstrating that *C. sakazakii* infection could induce severe intestinal inflammation in mice. Weng et al. [24] found that *C. sakazakii* infection caused damage throughout the intestines of newborn mice, with the jejunum being slightly damaged and the ileum being severely damaged. In this study, CD-1 mice infected with *C. sakazakii* at 3 days old were selected to construct the NEC model, and examined the regulatory effect of PhoP/PhoQ system on intestinal inflammation. The results showed that *C. sakazakii* infection caused growth retardation and severe intestinal inflammation in the neonatal mice (Figure 2). The H&E staining and pathology scores indicated that the neonatal mice infected with the WT strain and Δ*phoPQ^C^* strain exhibited more severe lesions than those infected with the Δ*phoPQ* strain, with the ileum showing the most serious damage, including intestinal epithelial shedding, villus structure destruction, and inflammatory cell infiltration (Figure 3A–C). These findings suggest that *C. sakazakii* causes severe intestinal injury in neonatal mice, and the deletion of the *phoPQ* gene reduces its pathogenicity (Figure 1, Figure 2 and Figure 3).

The main causes of NEC include prematurity, apoptosis, inflammation, and oxidative stress [32]. Apoptosis is a significant reason for the breakdown of intestinal mucosal barrier function [33]. The apoptosis process can be activated through various pathways, with caspase-3 playing a key role as an apoptosis executor [34]. Yang et al. [35] showed that *C. sakazakii* ATCC 29544-infected mice induced apoptosis of ileal cells by activating caspase-3 activity. Similarly, in this study, the *C. sakazakii* BAA-894 strain-infected neonatal mice activated caspase-3 activity. However, the caspase-3 activity in the Δ*phoPQ* group exhibited a significant decrease (*p* < 0.05) (Figure 4).

Inflammation is a protective response to cell infection or injury, mediated by the activation of various immune cells [36]. The symptoms of NEC encompass increased intestinal inflammation and elevated levels of cytokines [37]. Fan et al. demonstrated that *B. fragilis* inhibited *C. sakazakii*-induced NEC by regulating inflammation, cell apoptosis, and pyroptosis [38]. The secretion of TNF-α by various cells serves as a trigger and inducer for an inflammatory cascade [39]. IL-6 is an important immunomodulatory cytokine that promotes the migration, recruitment, and activation of inflammatory cells [40]. It has been reported that bacterial entry into tissues can cause severe inflammatory responses triggered by cytokines [41]. In this study, the ELISA results showed that the expression of TNF-α and IL-6 was significantly up-regulated in the neonatal mice infected with *C. sakazakii* compared to the control mice (*p* < 0.05). Compared to the WT and Δ*phoPQ^C^* groups, the levels of TNF-α and IL-6 in the ileum of the Δ*phoPQ* group exhibited a significant reduction (*p* < 0.05) (Figure 5A,B). Similarly, the qRT-PCR results showed that, compared with the PBS group, TNF-α expression in the WT, Δ*phoPQ*, and Δ*phoPQ^C^* groups was up-regulated by 5.51-, 1.70-, and 3.42-fold, respectively, and IL-6 expression was up-regulated by 4.85-, 1.22-, and 3.52-fold, respectively (Figure 6). Similar to TNF-α, IL-1β plays a crucial part in the host’s response to inflammation [42]. In this study, IL-1β transcription levels were significantly (*p* < 0.05) up-regulated in the ileum of the WT and Δ*phoPQ^C^* groups compared to the PBS and Δ*phoPQ* groups. However, the Δ*phoPQ* group did not exhibit a significant difference compared to the PBS group (Figure 6). These results suggest that the PhoP/PhoQ system affects the ability of *C. sakazakii* to infect the intestinal tract of newborn mice by regulating the gene expression of inflammatory factors.

Toll-like receptor 4 (TLR4) mediates inflammation by recognizing lipopolysaccharides in Gram-negative bacteria [43]. The NF-κB pathway represents a pivotal downstream pathway for all LPS-mediated signal transduction pathways, suggesting that the TLR4/NF-κB pathway may serve as a crucial target for inflammatory responses and organ damage [44,45]. The TLR4/NF-κB pathway is critical in the pathogenesis of NEC [46,47]. Activation of TLR4 induces NF-κB nuclear translocation and overexpression of proinflammatory cytokines, thereby participating in the pathogenesis of NEC [48,49,50]. Zhang et al. [48] demonstrated that β-glucan may exert inhibitory effects on intestinal inflammation by modulating the TLR4/NF-κB pathway, thereby preventing NEC in newborn mice. Liu et al. [51] demonstrated that *Lactobacillus reuteri* mitigates the incidence and severity of NEC through modulation of the TLR4/NF-κB pathway. In this study, both the mRNA transcription levels and protein levels of NF-κB p65 in the ileum of the mice infected by the WT and Δ*phoPQ^C^* strains exhibited a significant increase compared to those in the PBS group, while the expression levels of the Δ*phoPQ* group were similar to those in the control group (Figure 6 and Figure 7). Similarly, TLR4 mRNA and protein levels were significantly elevated in the WT- and Δ*phoPQ^C^*-infected mice compared to those in the Δ*phoPQ* group (Figure 7 and Figure 8). However, the protein levels of IκBα in the ileum of the *C. sakazakii*-infected mice were significantly decreased compared to the PBS group, with lower expression levels in the WT and Δ*phoPQ^C^* groups than in the Δ*phoPQ* group (Figure 8). Overall, the results suggest that *C. sakazakii* infection activates the TLR4/NF-κB pathway in the mouse ileum and may regulate inflammation mediated by this pathway through the PhoP/PhoQ system.

The PhoP/PhoQ system is critical in bacterial pathogenicity and antibiotic resistance, rendering it attractive for the development of novel antimicrobial agents. It has been reported that the PhoP/PhoQ systems in *Salmonella* have been targeted for screening quinazoline-based compounds with anti-virulence effects [52]. Carlos et al. [53] demonstrated that N′-(Thiophen-2-methyl-benzoylhydrazide) (A16B1) can effectively target the PhoP/PhoQ system by studying the antibacterial mechanism of A16B1 and a gentamicin protection assay, thereby mitigating the toxicity and drug resistance of *Salmonella*. Cai et al. [54] reported that targeting the PhoQ histidine kinase could serve as a promising therapeutic strategy against *Shigella*, effectively attenuating its pathogenicity. Although drugs targeting the PhoP/PhoQ system are still in the research phase, continued exploration of inhibitors against this system holds significant potential as a method for developing novel antimicrobial strategies, particularly for combating multidrug-resistant G-bacteria.

## 5. Conclusions

In summary, deletion of the *phoPQ* gene significantly reduced the pathogenicity of *C. sakazakii* in neonatal mice, including a reduced colonization ability in the ileum, reduced intestinal damage, decreased caspase-3 activity, and down-regulated levels of IL-1β, TNF-α, IL-6, NF-κB p65, and TLR4. In addition, *C. sakazakii* activates the TLR4/NF-κB pathway in the mouse ileum, and the deletion of the phoPQ gene may mitigate the inflammatory response mediated by this pathway. These findings suggest that the PhoP/PhoQ system plays a pivotal role in regulating the pathogenicity of *C. sakazakii* within the neonatal mouse intestinal tract.

## Figures and Tables

**Table 1:** Primers used for qRT-PCR.

Gene	Sequence (5′-3′)	References
IL-1β	F: GCAACTGTTCCTGAACTCAACT R: ATCTTTTGGGGTCCGTCAACT	[25]
TNF-α	F: CCCTCACACTCAGATCATCTTCT R: GCTACGACGTGGGCTACAG	[26]
IL-6	F: TAGTCCTTCCTACCCCAATTTCC R: TTGGTCCTTAGCCACTCCTTC	[26]
NF-κB p65	F: AGGCTTCTGGGCCTTATGTG R: TGCTTCTCTCGCCAGGAATAC	[27]
TLR4	F: ATGGCATGGCTTACACCACC R: GAGGCCAATTTTGTCTCCACA	[28]
GAPDH	F: AGGTCGGTGTGAACGGATTTG R: TGTAGACCATGTAGTTGAGGTCA	

## Data Availability

The original contributions presented in the study are included in the article, further inquiries can be directed to the corresponding author.
